# Supplementary material for: Imaging Cu2O nanocube hollowing in solution by quantitative in situ X-ray ptychography
Source: Nat Commun. 2022 Aug 29;13:4971. doi: 10.1038/s41467-022-32373-2 (PMC9424245; doi:10.1038/s41467-022-32373-2)
Supplement: Supplementary file 1 — Supplementary Information [file 41467_2022_32373_MOESM1_ESM.pdf]

Supplementary Information

Imaging Cu_2O nanocube hollowing in solution by quantitative in situ X-ray ptychography

Author list: Lukas Grote[1,2], Martin Seyrich[1,2], Ralph Döhrmann[2], Sani Y. Harouna-Mayer[1,4], Federica Mancini[1,5], Emilis Kaziukenas[1,6], Irene Fernandez-Cuesta[3,4], Cecilia A. Zito[1,7], Olga Vasylieva[1], Felix Wittwer[1,2], Michal Odstrčzil[8,‡], Natnael Mogos[1], Mirko Landmann[2], Christian G. Schroer[1,2,9], and Dorota Koziej[1,4,*]

Affiliations:
[1] University of Hamburg, Institute for Nanostructure and Solid-State Physics, Center for Hybrid Nanostructures, Luruper Chaussee 149, 22761 Hamburg, Germany
[2] Center for X-ray and Nano Science CXNS, Deutsches Elektronen-Synchrotron DESY, Notkestraße 85, 22607 Hamburg, Germany
[3] University of Hamburg, Department of Physics, Luruper Chaussee 149, 22761 Hamburg, Germany
[4] The Hamburg Centre for Ultrafast Imaging, Hamburg, Germany
[5] Institute of Science and Technology for Ceramics (ISTEC), National Research Council (CNR), Via Granarolo 64, 48018 Faenza (RA), Italy
[6] Department of Applied Mathematics and Theoretical Physics, University of Cambridge, Wilberforce Road, Cambridge CB3 0WA, United Kingdom
[7] São Paulo State University UNESP, Rua Cristóvão Colombo 2265, 15054000 São José do Rio Preto, Brazil
[8] Paul Scherrer Institute, Forschungsstrasse 111, 5232 Villigen PSI, Switzerland
[9] Helmholtz Imaging Platform, Deutsches Elektronen-Synchrotron DESY, Notkestraße 85, 22607 Hamburg, Germany
[‡]Present address: Carl Zeiss SMT, Carl-Zeiss-Straße 22, 73447, Oberkochen, Germany
* Correspondence to: dorota.koziej@physnet.uni-hamburg.de

Supplementary Note 1. Technical information on the in situ reactor

A schematic representation of our in situ reactor in the form of an exploded view is shown in Supplementary Figure 1. The liquid container of the reactor is a PTFE autoclave with two 125 µm thin polyimide windows on opposite sides for high X-ray transparency. The autoclave has a volume of 2 mL to ensure undisturbed reaction kinetics as compared to laboratory vials. The autoclave is placed between flat heating elements with a curved surface, pushing onto the flexible polyimide windows at the center of the container which takes a concave shape. This way, the beam path through the liquid measures only 1 mm for low attenuation and background, while at the same time a large liquid volume is provided. To ensure position stability within the single-digit nanometer range, the heatable body is made of Invar and titanium for low thermal expansion. The body is thermally isolated from the base attached to the nano-positioning stage by a Zerodur (Schott AG, Mainz, Germany) part with almost no thermal expansion and low thermal conductivity. The base is actively cooled with an air flow through a copper pipe. The heating elements are current-regulated and operated with DC to avoid vibrations induced by switching. A PID-controller provides high temperature stability of ±0.05 °C.

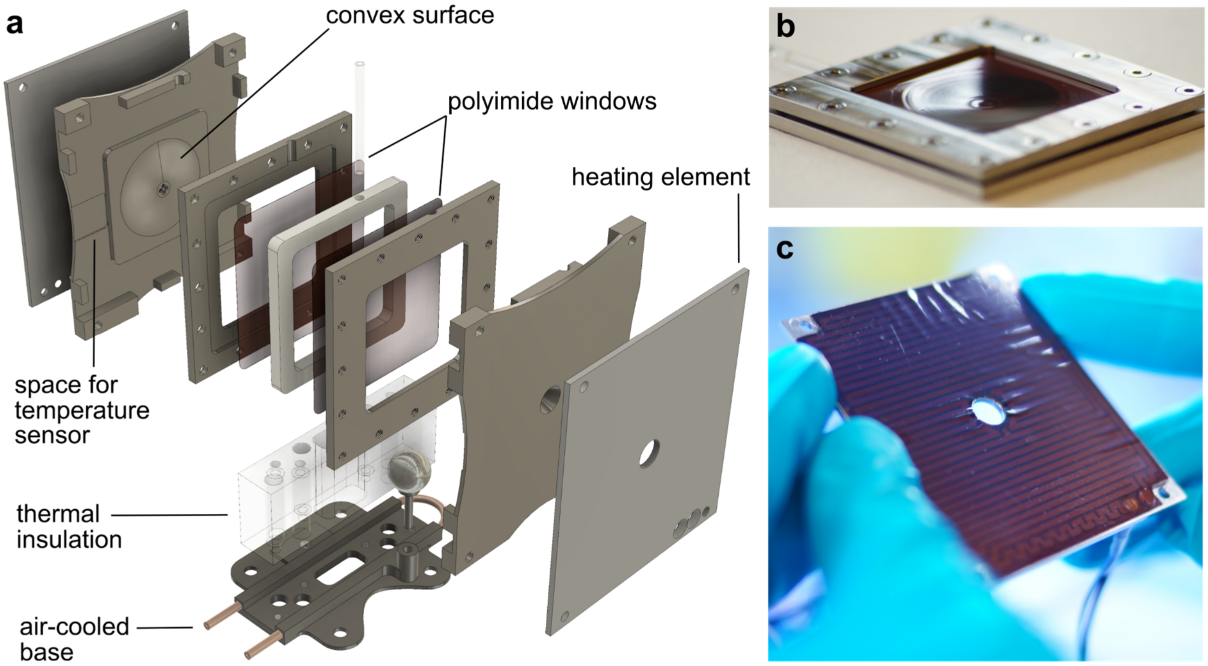

Supplementary Figure 1. Schematic representation of the in situ reactor. a The exploded view of the in situ reactor shows all components individually. The liquid container is made of a PTFE frame covered with two polyimide windows and clamped between two Invar frames. The container is placed between two Invar heating covers with a convex surface. Flat heating elements provide uniform heat from two sides. A thermal insulation made of Zerodur is placed between the heated body and the air-cooled base. **b** Photograph of the liquid container after a reaction at 155 °C. The concave shape results in a window distance of 1 mm at the center of the container. **c** Photograph of a resistive heating element made of titanium and flexible circuits printed on polyimide.

To verify the position stability, we conducted thermal simulations of the reactor applying the finite-element method using the software ANSYS Mechanical version 17.2 (ANSYS Inc, Canonsburg, USA). The results are summarized in Supplementary Figure 2. Part a of the figure shows the temperature field in the steady state across the reactor when the reaction temperature of 155 °C is reached. We find that the squared upper body is evenly heated (red), while the thermal insulation efficiently keeps the heat away from the base (blue). Due to the large temperature gradient across the insulation, it is important that we used Zerodur with extremely low thermal expansion for this part.

We also simulated the expected residual thermal drifts of the imaged region of the reactor given a temperature instability of ±0.05 °C. By Fourier-transforming a measured temperature profile of the reactor at the target temperature, we identified that the main frequency component of the residual temperature fluctuations has a period of 353 s. We used a sinusoidal temperature profile with this period for a transient simulation, tracking the variation in the vertical distance from the base to the beam position on the reactor. Due to the symmetric design of the reactor, we expect no significant thermal drift in the horizontal direction. Supplementary Figure 2b shows the thermal expansion as obtained from the transient simulation. We find a residual vertical drift below ±8 nm. The actual drifts during data acquisition may have been larger due to thermal movement of the polyimide windows not covered by the simulation.

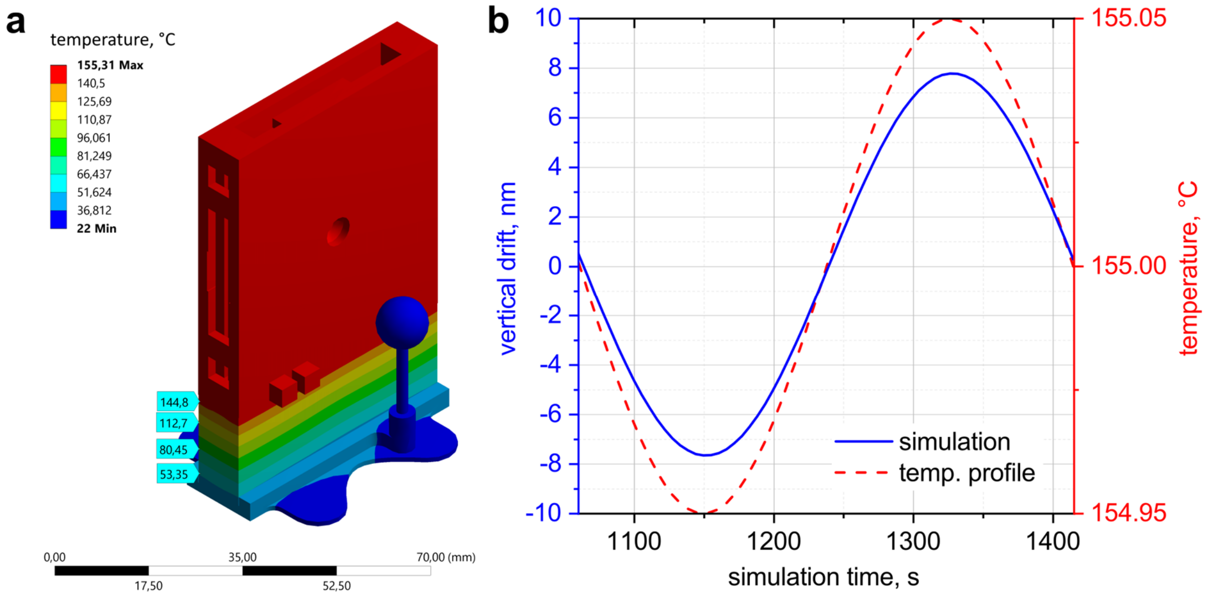

Supplementary Figure 2. Thermal simulations on the in situ reactor. a Simulation of the heat distribution over the assembled reactor in the stationary case at 155 °C. The upper squared body of the reactor containing the reaction solution is evenly heated. The temperature drops off across the thermal insulation, keeping the base at a low temperature between 30 and 50 °C. **b** Transient simulation of the vertical expansion between the base and the beam position. A sinusoidal temperature profile with a period of 353 s was applied. The residual drift is below ±8 nm.

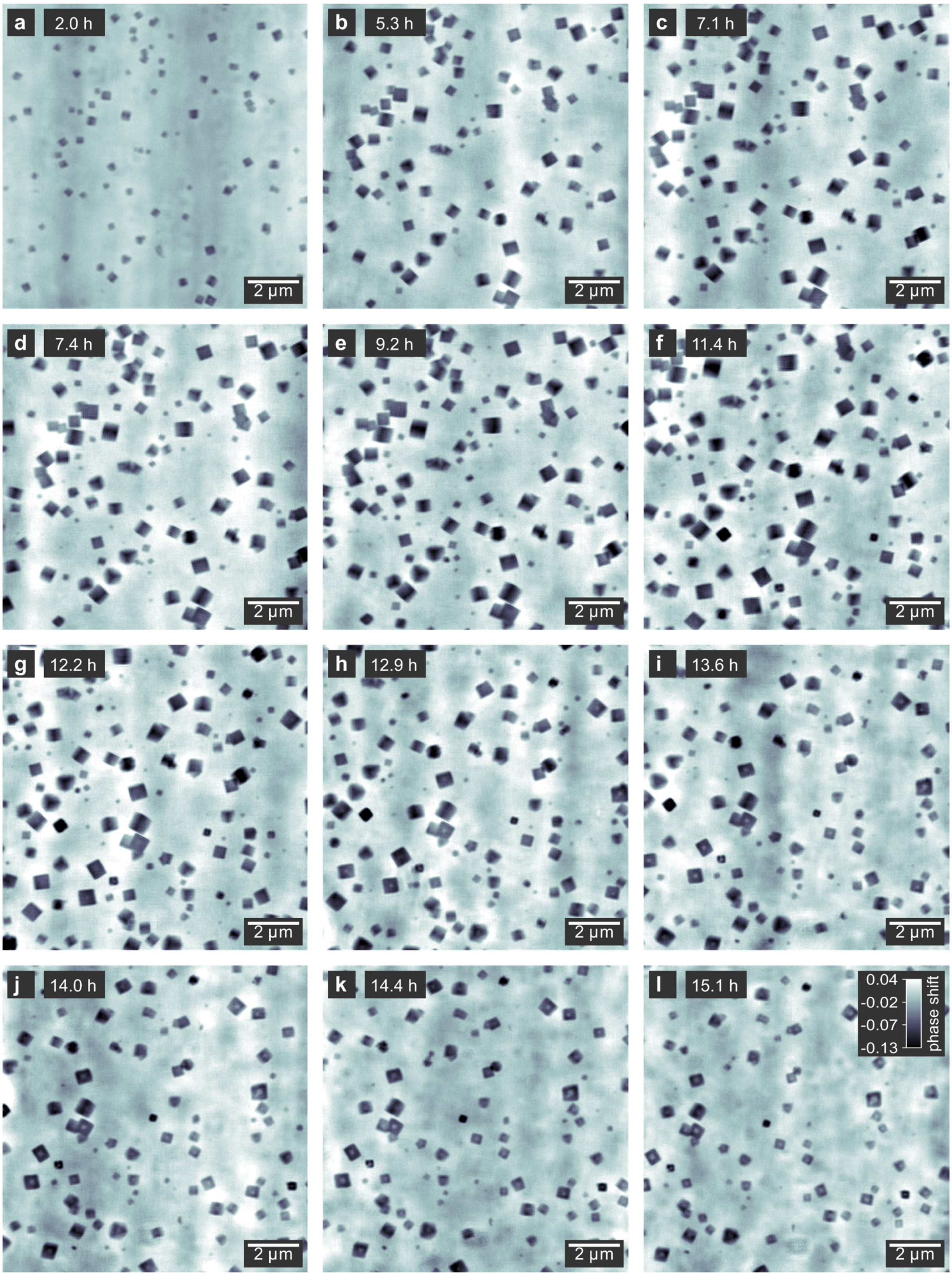

Supplementary Figure 3. Overview of the reaction on the exit window. a-l Ptychographic reconstructions of nanocubes on the exit window of the in situ reactor. The gray scale indicates the phase shift of the images. At all reaction times, the particles are similar in size and morphology the those on the entrance window.

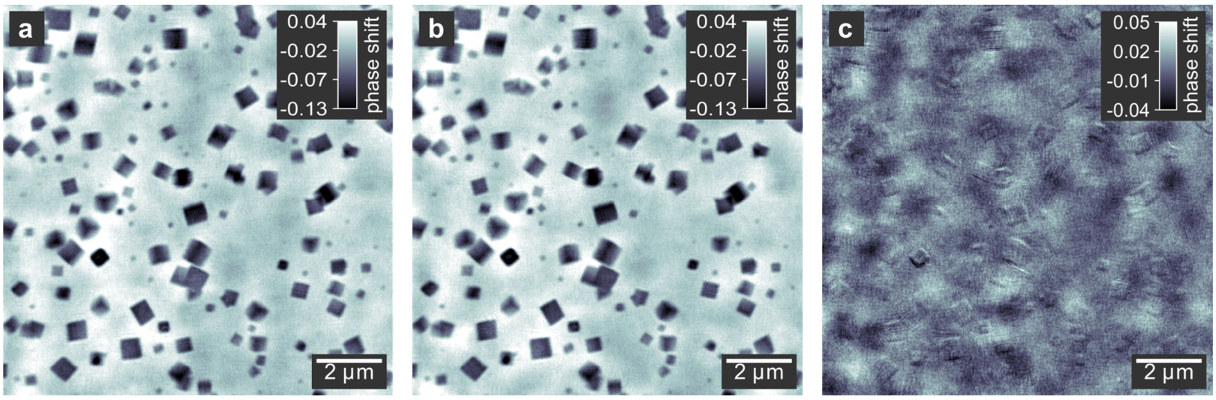

Supplementary Figure 4. Reconstructions from split data set. a-b Example of ptychographic reconstructions of nanocubes on the exit window of the reactor, each reconstructed with the data from half of the scan points compared to the full data set. These reconstructions were used to calculate the Fourier ring correlation of the exit window shown in Figure 2f. **c** Difference between the reconstructions in **a** and **b**. The gray scale indicates the phase shift of the images.

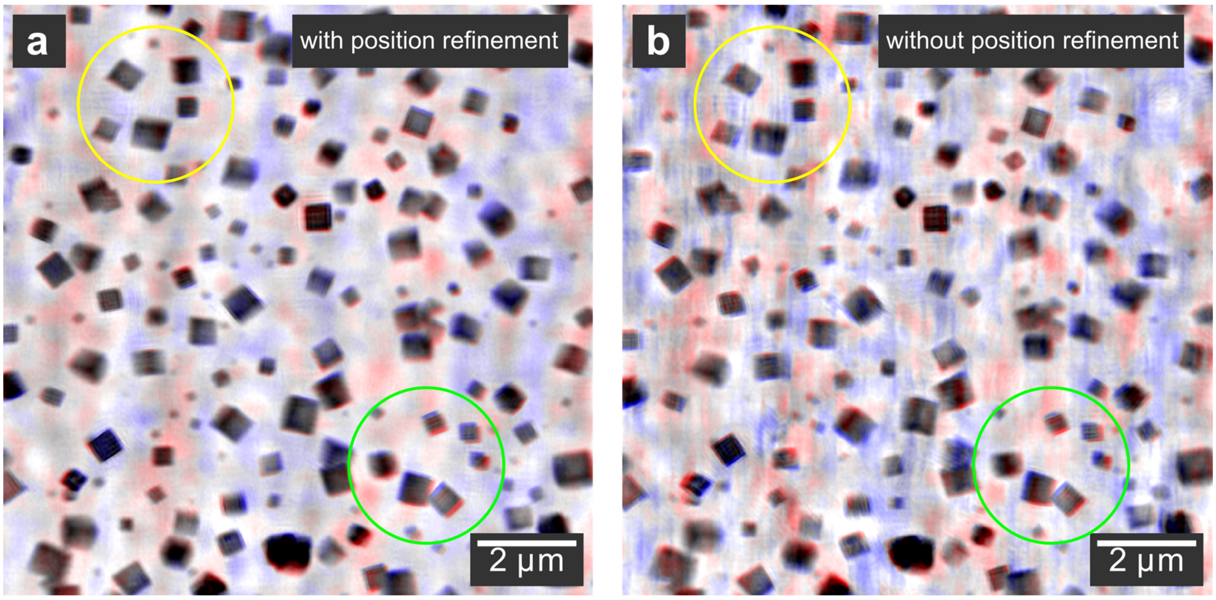

Supplementary Figure 5. Effect of position refinement during reconstruction. False-colored overlay of two consecutive ptychographic reconstructions from the entrance window of the reactor, reconstructed **a** with and **b** without position refinement[1] enabled during iterative phase retrieval. The overall quality of the reconstruction without position refinement is significantly worse due to thermal movements of the reactor during scanning not being corrected for. Blue and red shadows at particle edges indicate an instability in the position of the respective particles between consecutive images, even though the images have been aligned to each other using the Scale-invariant Feature Transform (SIFT) method[2] implemented within the image analysis software ImageJ[3] (version 1.53k). From the particles highlighted with a yellow circle, it is visible that the position refinement stabilized their location, since more pronounced red and blue edges can be seen in **b** compared to **a**. Still, for other particles such as those highlighted with the green circle, a displacement remains irrespective of position refinement.

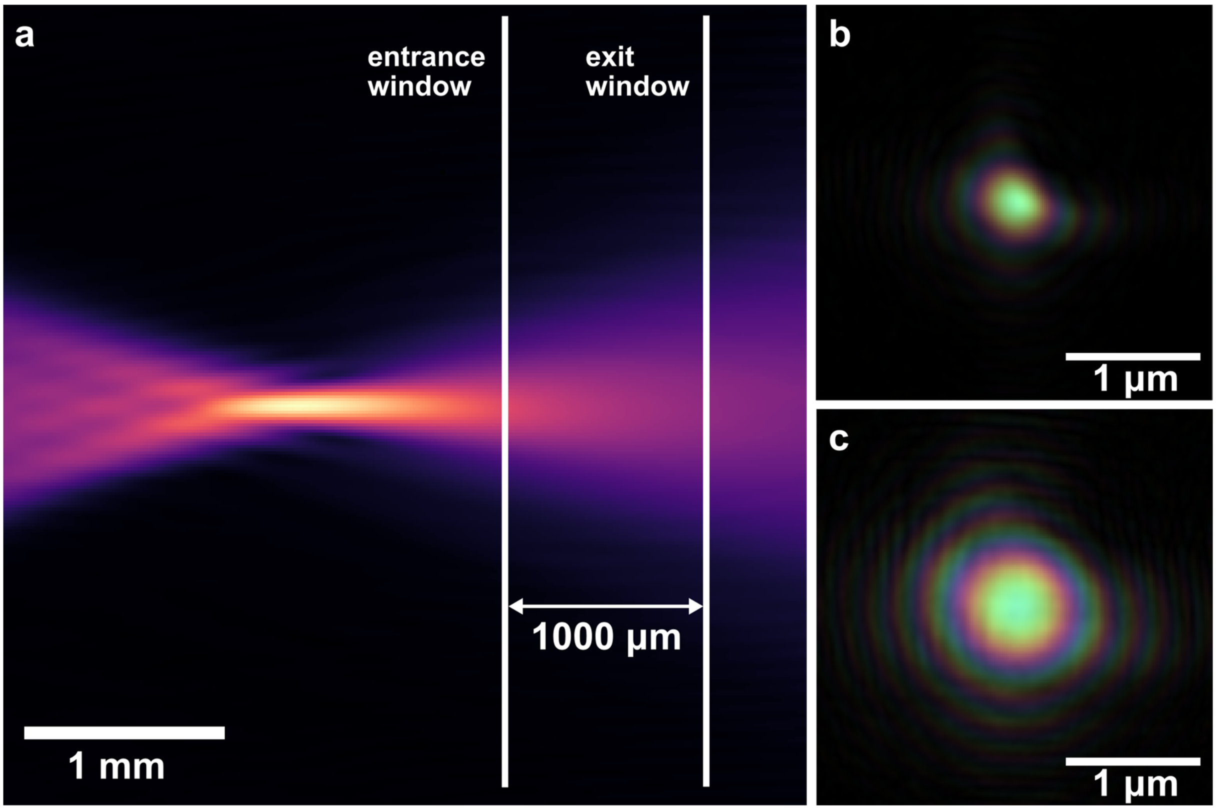

Supplementary Figure 6. Reactor position and illumination profiles. a Positions of the reactor windows along the caustic of the focused beam, displayed as the horizontal intensity. **b**-**c** Display of the illuminations on the entrance and exit windows, respectively. Here, brightness represents the amplitude and color represents the phase of the wavefield.

Supplementary Note 2. Distance between reactor windows

The multi-slice model[4, 5] allows to separately reconstruct several objects stacked along the beam direction from a single ptychographic data set. To this end, the distance between the layers must be known. In Supplementary Figure 7, we exemplarily show the effect of a wrong distance applied to the multi-slice model during reconstruction. Parts a and c of the figure show reconstructions with the correct distance set for the entrance and exit windows, respectively, while parts b and d show reconstructions with a wrong distance setting. We note that for the entrance window, the wrong distance results in artefacts in the form of fringes and additional background fluctuations. The exit window is the second layer in the multi-slice model and its reconstruction is initialized after 20 iterations. Here, a wrong distance leads to a loss in resolution and thus to a blurred image.

Directly after the assembly of the reactor, the distance between the polyimide windows is 1 mm, however, we observed that at 155 °C and in contact with the solvent, the windows deform over time and the distance between them shrinks. Therefore, we track the distance during the in situ imaging (Supplementary Figure 7e). By visual inspection of reconstructions using different possible distances, we evaluate the correct distance several times, as indicated by red arrows in Supplementary Figure 7e. Between the evaluations, we interpolate the distance. To confirm that the visual inspection results in an accurate determination of the window distance, we exemplarily evaluate the resolution of the exit window reconstruction at many possible distances for the image taken after 510 min reaction time (Supplementary Figure 7f). This procedure indicates an optimal distance of 799 µm, which is in good agreement with the distance of 760 µm found by visual inspection.

The separation of objects using the multi-slice model only works as long as their distance is larger than the depth of field (DOF) of the imaging setup, in this case 280 µm according to Equation 1. This is why starting at 15 h reaction time, when the window distance gets close to the DOF, the layer separation starts to produce artefacts and it fails after 16.7 h reaction time. From this time on, we can only reconstruct an overlay of nanocubes on both windows.

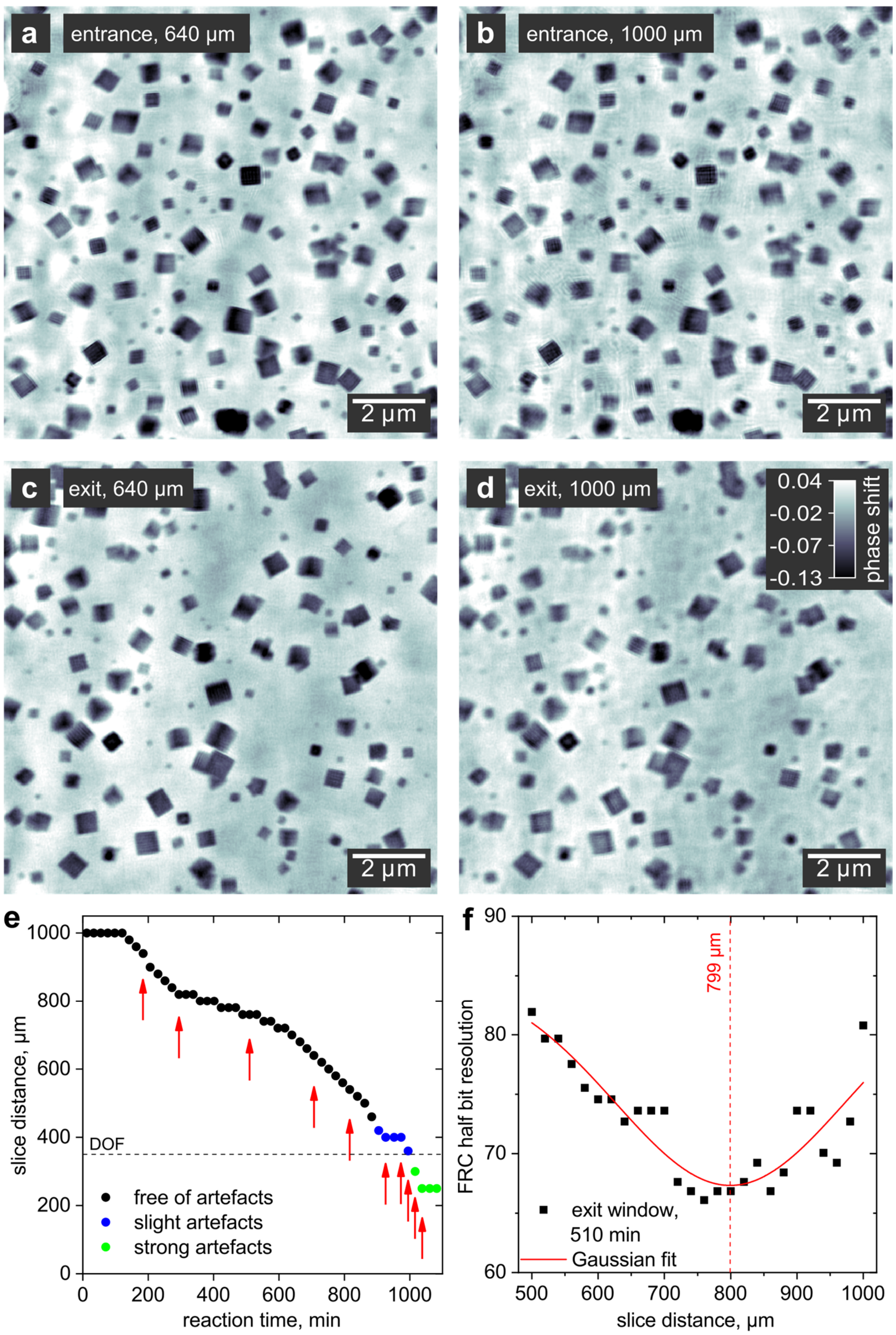

Supplementary Figure 7. Finding distance between reactor windows. a-b Example ptychographic reconstructions of nanocubes on the entrance window of the reactor applying the correct distance of 640 µm and a wrong distance of 1000 µm, respectively. **c-d** Ptychographic reconstructions from the exit window in accordance to **a-b**. The gray scale indicates the phase shift of the images. **e** Distances between the reactor windows for all time steps of the in situ experiment. Red arrows mark the times for which the distance was determined by eye inspection. **f** Slice distance determined by examining the Fourier ring correlation[6, 7] (FRC) resolution of the exit window reconstructions, exemplarily performed for 510 min reaction time. The Gaussian fit indicates that the best resolution is obtained for a distance of 799 µm, which agrees with the distance found by visual inspection in **e**.

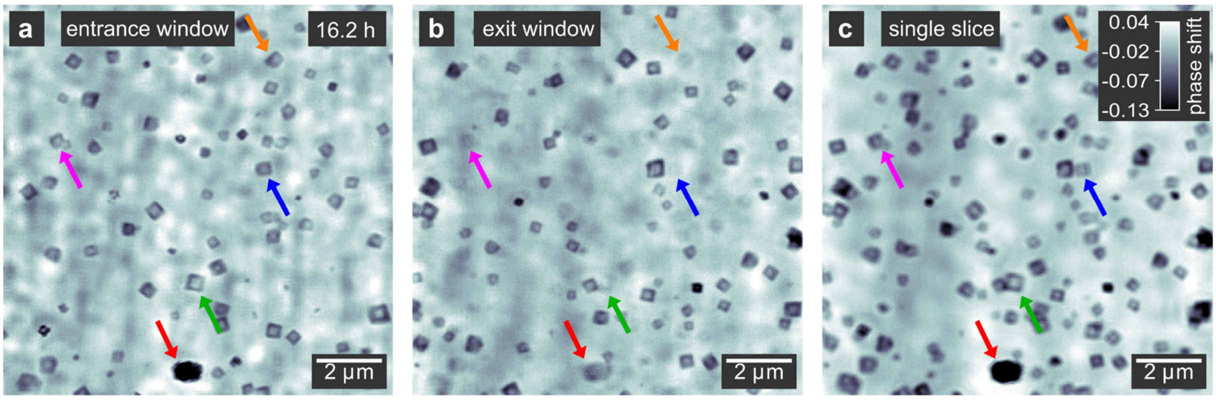

Supplementary Figure 8. Effect of incomplete slice separation. a-**b** Ptychographic image slices corresponding to the entrance and exit windows of the reactor, respectively, obtained from a multi-slice reconstruction. The images were obtained after 16.2 h reaction time, when the distance between the two windows had decreased to 400 µm due to deformation of the polyimide foils. Since this distance is not much larger than the DOF of 280 µm of the imaging experiment (see Supplementary Notes 2), the slice separation is incomplete. Colored arrows indicate particles in the entrance window slice (**a**) which are also dimly visible in the exit window slice (**b**). For comparison, **c** shows the corresponding reconstruction with only one object layer (single-slice). The gray scale indicates the phase shift of the images.

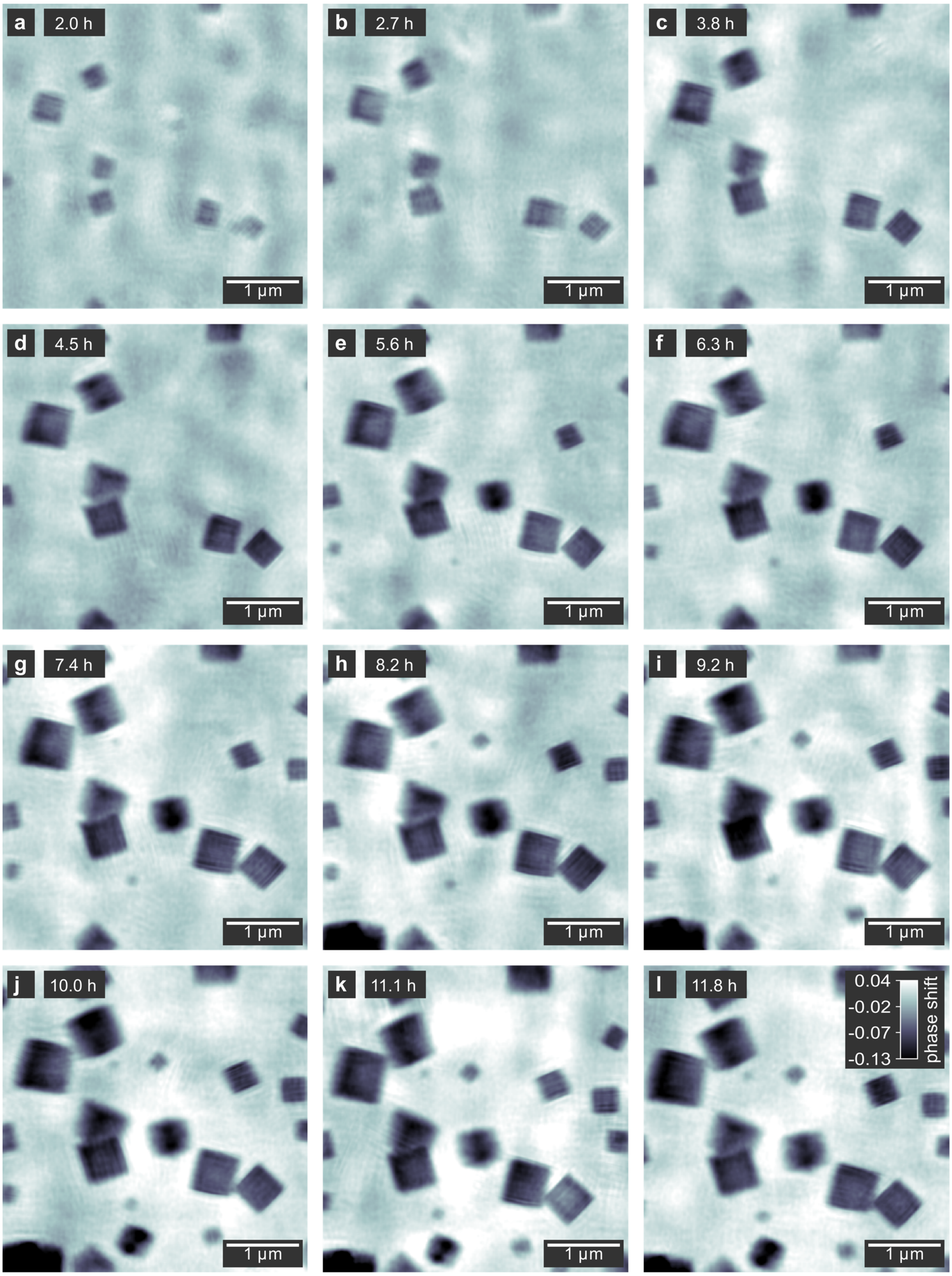

Supplementary Figure 9. Detailed view of the growth phase of Cu_2O nanocubes a-l Ptychographic reconstructions of the entrance window of the reactor with a reduced field of view. The gray scale indicates the phase shift of the images.

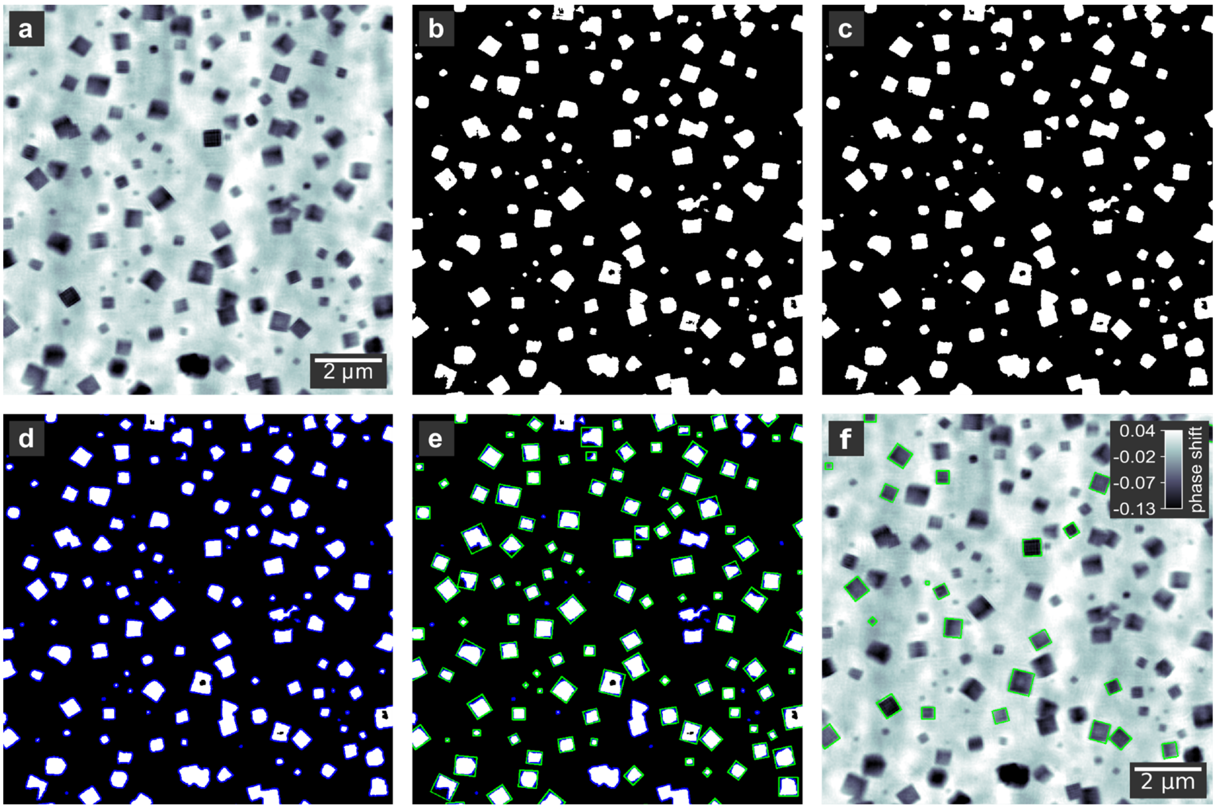

Supplementary Figure 10. Overview of the image processing steps for tracking nanocubes with the OpenCV library.[8] **a** Original ptychographic phase image. **b** Image after applying an adaptive threshold. **c** Image after applying a closing step. **d** Contours in the binary image highlighted in blue. **e** For each contour, the smallest rectangle that contains all points of the contour is drawn in green. **f** From the image in **e**, only squared shapes are selected and overlayed with the original phase image. The identified particle dimension match well. The gray scale indicates the phase shift of the image.

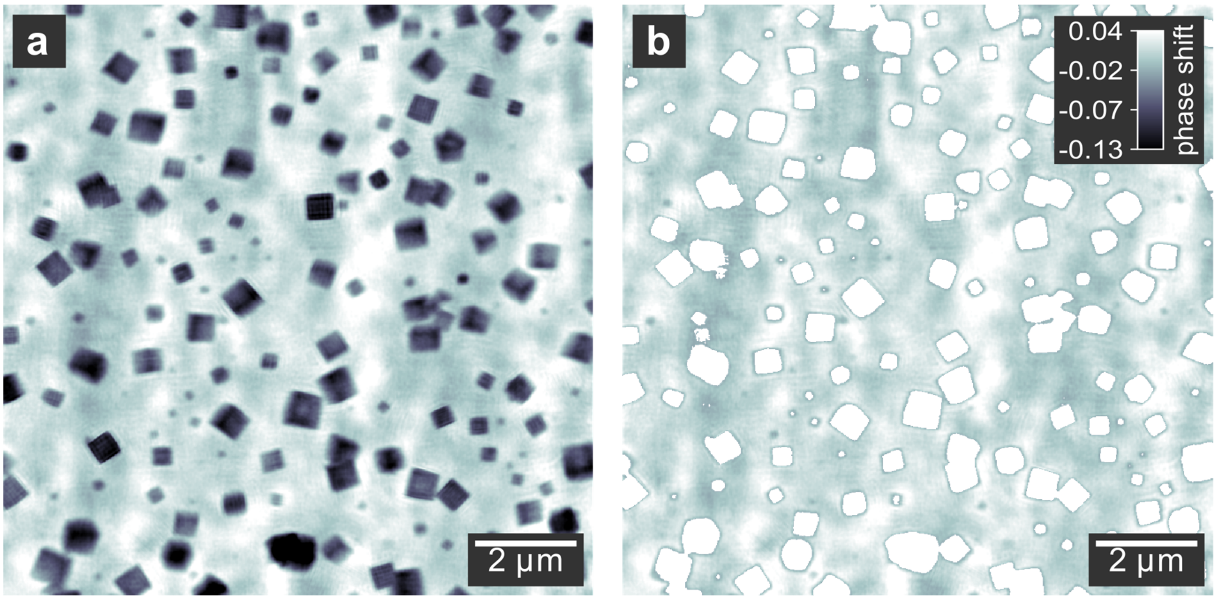

Supplementary Figure 11. Background selection. a Original ptychographic phase image. **b** Image after applying a phase threshold of -0.02 to select only the background. The gray scale indicates the phase shift of the images.

Supplementary Note 3. Error calculation for the aspect ratio of the nanocuboids

The error Δa of the aspect ratio results from the error propagation of the in-plane particle size and the out-of-plane thickness. We calculate Δa according to

$$\Delta a = a\left(\frac{\Delta\phi}{\phi} + \frac{\Delta d}{d}\right) \quad \text{(S1)}$$

where d and a are the edge length and the aspect ratio of a nanocuboids, respectively, ϕ is the mean absolute phase shift measured for a specific nanocuboid, Δd is the error of the edge length given by the spatial resolution of the ptychographic reconstructions, and $\Delta\phi$ is the error of the phase shift given by the standard deviation of all pixel values within an area covering 70% of the full particle dimension (blue rectangles in Figure 4a).

We calculate the error of the aspect ratio individually for each tracked nanocuboids in each image. For clarity, in Figure 4b and Supplementary Figure 12 the error bar indicates the mean of all calculated errors, equaling 0.165. For all tracked particles at all reaction times, the highest individual error equals 0.3.

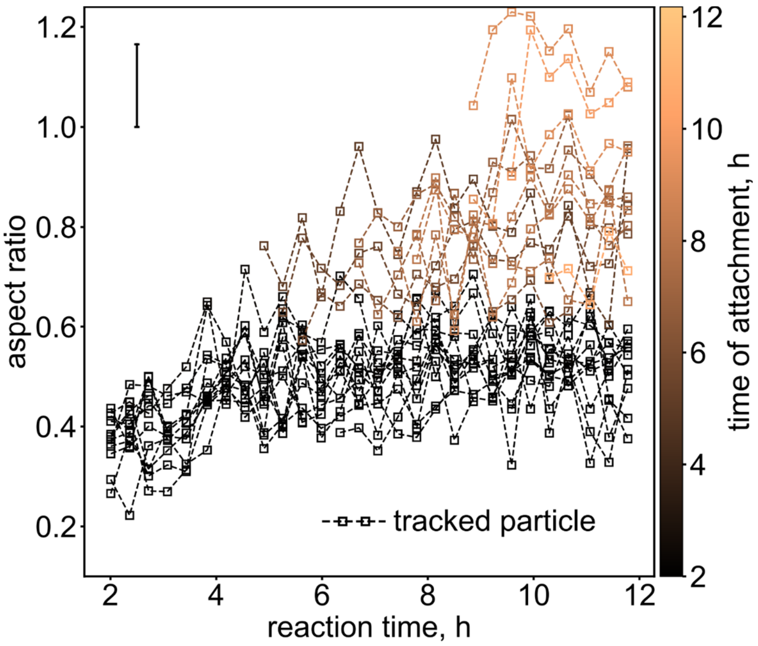

Supplementary Figure 12. Evolution of the aspect ratio for all tracked particles highlighted in Figure 4a. The aspect ratio is calculated as the quotient of the in-plane dimension and the out-of-plane thickness of the nanocuboids. Yellow color indicates the time when a particle attaches to the substrate. No moving average is applied to this data. The later the attachment takes place during the growth phase, the more cubic the respective particle is. The error bar represents the mean error resulting from the error propagation of the in-plane particle size and the out-of-plane thickness of all tracked particles.

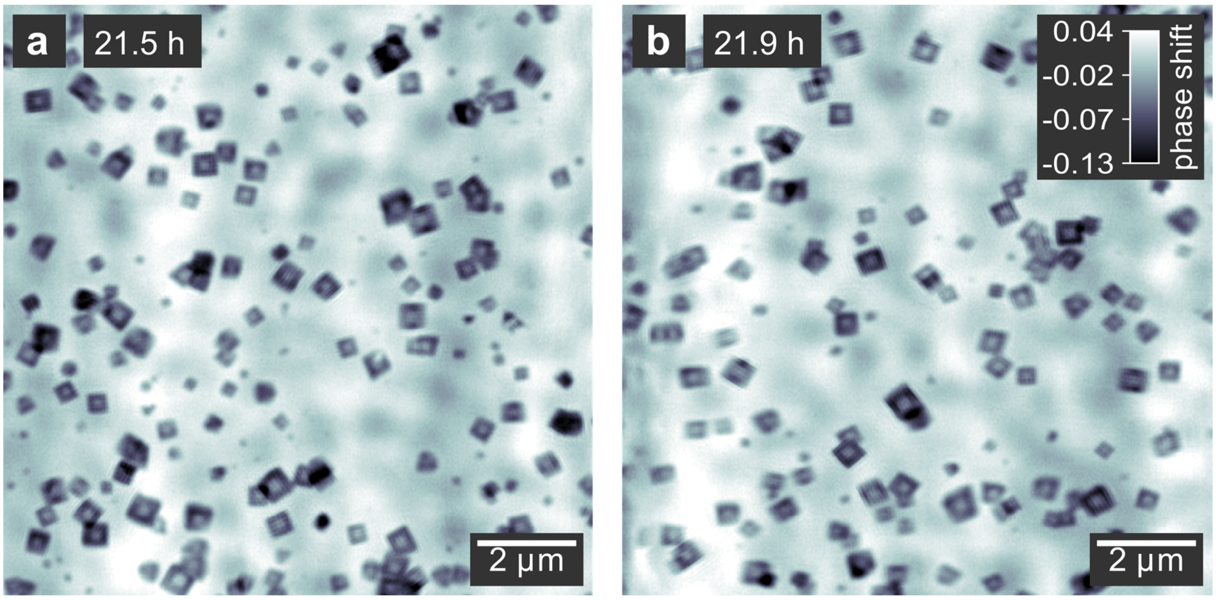

Supplementary Figure 13. Confirmation of void formation without beam. Ptychographic reconstructions of nanocubes at the end of the void formation. The images in **a** and **b** were taken at a different part of the reactor window, ca. 2 mm away from the previously exposed region, confirming that the hollowing process is not triggered by the X-ray beam. The images show overlays of particles on both reactor windows since multi-slicing could not be applied due to the decreasing distance between the windows. The gray scale indicates the phase shift of the images.

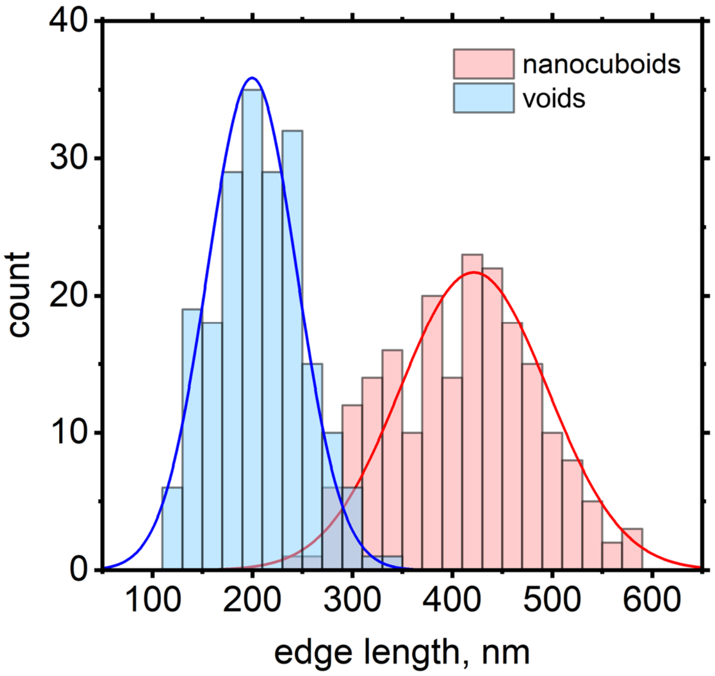

Supplementary Figure 14. Histogram of outer dimensions and void sizes of the nanocuboids. Here, we display the same data that is shown in Figure 5j, but in the form of a histogram to recognize the size distributions. The analysis is based on 200 nanocuboids.

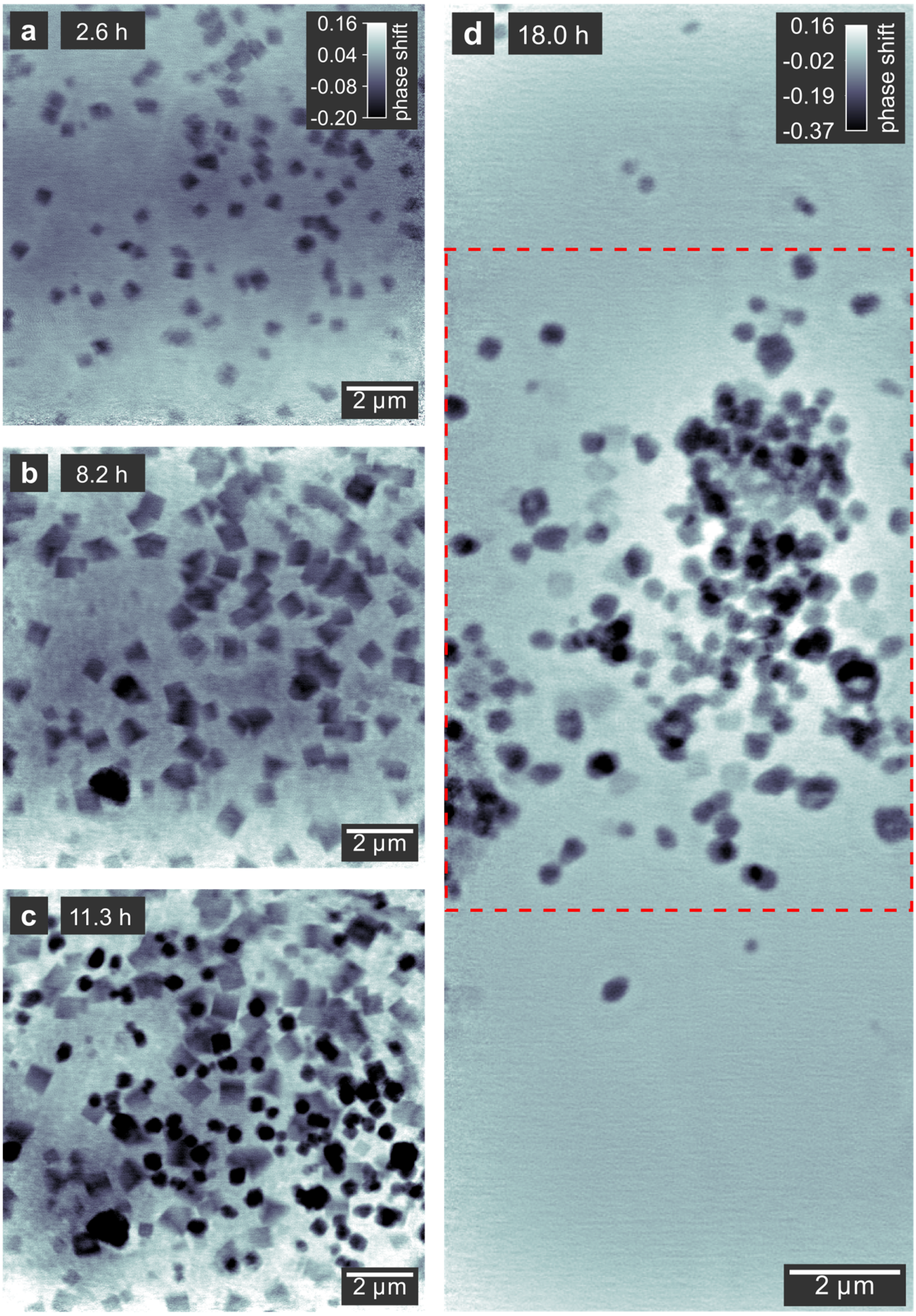

Supplementary Figure 15. Overview of the in situ imaging experiment at a photon energy of 8.98 keV. The photon energy was set just above the K edge of copper. **a**-**c** Ptychographic reconstructions of nanocubes overlayed on both windows of the in situ reactor. **d** Ptychographic reconstruction with a larger field of view, highlighting the previously imaged region from **a**-**c** with a red rectangle. The X-ray beam triggered the nucleation and attachment of nanoparticles at the reactor windows. Almost no particles are found outside the illuminated area after 18 h reaction time. The gray scale indicates the phase shift of the images.

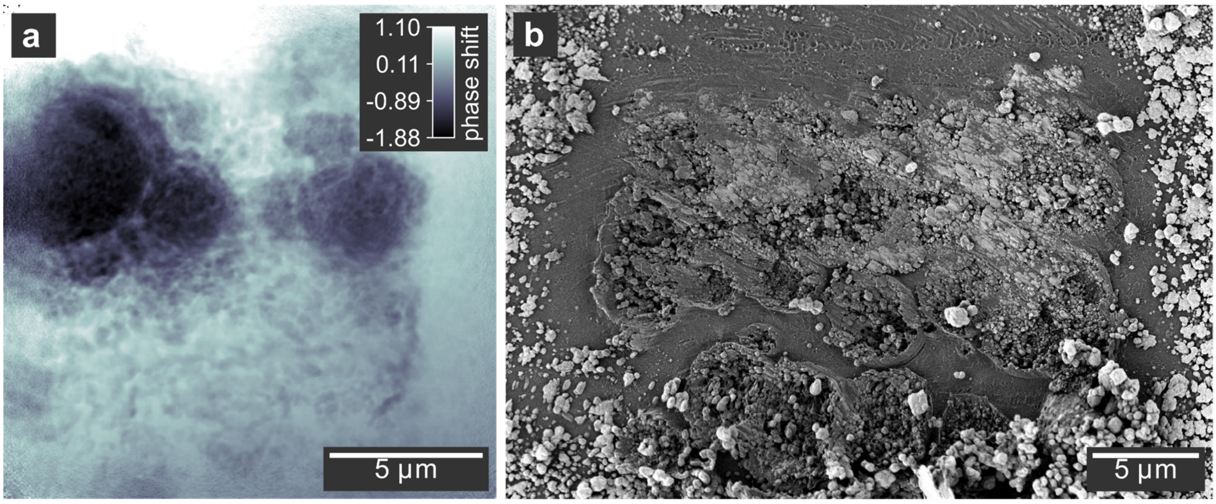

Supplementary Figure 16. Beam-induced deviation of the reaction imaged at a photon energy of 8.98 keV. a Ptychographic reconstruction and **b** SEM image of the damaged region. Micrometer-sized spherical structures formed under illumination instead of the expected nanocuboids. The gray scale indicates the phase shift of the ptychographic image.

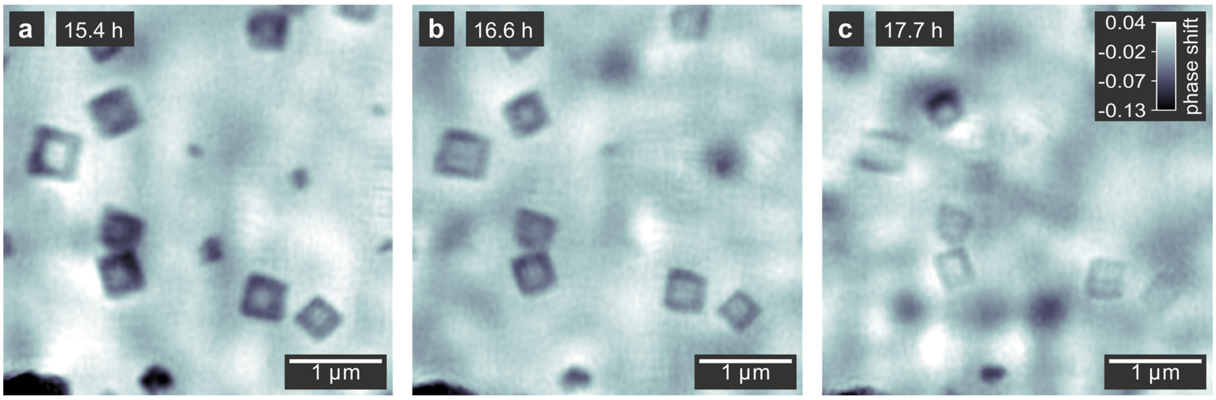

Supplementary Figure 17. Hollow metallic Cu nanocuboids vanishing under X-ray exposure at 15.25 keV. a-**c** Ptychographic reconstructions of nanocubes growing on the entrance window of the in situ reactor. We observe that after the end of the void formation, the particles shrink mostly in thickness as evident from the lower phase shift at later reaction times. Also, the outer dimension of the nanocuboids shrink to a lower extent. The gray scale indicates the phase shift of the ptychographic image.

Supplementary References

1. Maiden, A. M., Humphry, M. J., Sarahan, M. C., Kraus, B. & Rodenburg, J. M. An annealing algorithm to correct positioning errors in ptychography. *Ultramicrosc.* **120**, 64-72 (2012).
2. Lowe, D. G. Distinctive image features from scale-invariant keypoints. *Int. J. Comp. Vis.* **60**, 91-110 (2004).
3. Schneider, C. A., Rasband, W. S. & Eliceiri, K. W. NIH Image to ImageJ: 25 years of image analysis. *Nat. Methods* **9**, 671-675 (2012).
4. Tsai, E. H., Usov, I., Diaz, A., Menzel, A. & Guizar-Sicairos, M. X-ray ptychography with extended depth of field. *Opt. Expr.* **24**, 29089-29108 (2016).
5. Maiden, A. M., Humphry, M. J. & Rodenburg, J. M. Ptychographic transmission microscopy in three dimensions using a multi-slice approach. *J. Opt. Soc. Am. A* **29**, 1606-1614 (2012).
6. Banterle, N., Bui, K. H., Lemke, E. A. & Beck, M. Fourier ring correlation as a resolution criterion for super-resolution microscopy. *J. Struct. Biol.* **183**, 363-367 (2013).
7. van Heel, M. & Schatz, M. Fourier shell correlation threshold criteria. *J. Struct. Biol.* **151**, 250-262 (2005).
8. Bradski, G. The OpenCV library. *Dr Dobb's J. Software Tools* **25**, 120-125 (2000).
