# Supplementary material for: Imaging Cu2O nanocube hollowing in solution by quantitative in situ X-ray ptychography
Source: Nat Commun. 2022 Aug 29;13:4971. doi: 10.1038/s41467-022-32373-2 (PMC9424245; doi:10.1038/s41467-022-32373-2)
Supplement: Supplementary file 3 — Description of Additional Supplementary Information [file 41467_2022_32373_MOESM3_ESM.pdf]

Description of Additional Supplementary Information

Title: Supplementary Movie 1

Description: Growth and hollowing of Cu_2O nanocuboids imaged in situ with X-ray ptychography.
